# Hindoo Lithotomy

**Published:** 1859-09

**Authors:** 


HINDOO LITHOTOMY.—The following lucid description, says the *Dublin Medical Press*, quoting from Hessler's

Hindoo Medicine, of the operation, (probably written one thousand years before our era) which was well adapted to the spare habits of the Asiatic, has a marked resemblance to that described by Celsus, who may have obtained it from the East, and has since been well known as cutting on the "gripe," and is interesting from being the most ancient on record:—"The surgeon is to press the calculus downward, by rubbing the abdomen with his right hand, while the middle and index fingers of his left, with the nails cut short, and well oiled, are inserted into the rectum. With well-regulated force the calculus is to be pushed downward, until it forms a swelling on the left side of the perineum, when it is to be cut open and removed. The surgeon must take care to remove the calculus entire; and with this object, a scoop is to be used, when it is soft, as when a small portion is left, it will slowly form another."

